# Synthesis, Characterization and Biological Evaluation of Metal Adamantyl 2-Pyridylhydrazone Complexes

**DOI:** 10.3390/molecules25112530

**Published:** 2020-05-29

**Authors:** Ihsan A. Shehadi, Fatima-Azzahra Delmani, Areej M. Jaber, Hana Hammad, Murad A. AlDamen, Raed A. Al-Qawasmeh, Monther A. Khanfar

**Affiliations:** 1 Department of Chemistry, Faculty of Sciences, University of Sharjah, Sharjah 27272, UAE; ishehadi@sharjah.ac.ae (I.A.S.); ralqawasmeh@sharjah.ac.ae (R.A.A.-Q.); 2 Department of Biology, Faculty of Science, Jerash University, Jerash 26150, Jordan; 3 Department of Chemistry, Faculty of Science, The University of Jordan, Amman 11942, Jordan; areejmajed@ju.edu.jo (A.M.J.); maldamen@ju.edu.jo (M.A.A.); 4 Department of Biology, Faculty of Science, The University of Jordan, Amman 11942, Jordan; hhammad@ju.edu.jo

**Keywords:** 2-pyridyl adamantanhydrazone, hydrazone complexes, antitumor, crystal structure

## Abstract

Four new complexes derived from adamantly containing hydrazone (**APH**) ligand with Cu(II) (**1**), Co(II) (**2**), Ni(II) (**3**) and Zn(II) (**4**), have been synthesized and characterized using different physicochemical methods. The structure of the ligand **APH** and its copper complex **1** have been established by single-crystal X-ray diffraction direct methods, which reveal that complex **1** has distorted square-pyramidal geometry. Complexes **1**–**4** are screened against seven human cancer cell lines namely, breast cancer cell lines (MCF7, T47D, MDA-MB-231), prostate cancer cell lines (PC3, DU145) and the colorectal cancer cell line Coco-2, for their antiproliferative activities. Complex **1** has shown a promising anticancer activity compared to the other ones. The structural and spectroscopic analysis of **APH** and its complexes are confirmed by DFT calculations.

## 1. Introduction

The potential of having complexes as anti-cancer agents stemmed from the accidental discovery of the cis Platine, *cis*-[Pt(II)(NH_3_)_2_Cl_2_], in the 1960s [1]. However, due to its toxicity and narrow selectivity, the search for more selective and potent complexes has exponentially increased since then, and a new era of complex research has evolved [2,3]. In the last decades, the search was directed towards finding platinum-based complexes, which are less toxic, with a better and wider range of selectivity. As a result of such hunt, a wide range of platinum complexes were introduced, like Pt(II) cisplatin, carboplatin, oxaliplatin and Pt(IV) satraplatine and diazides [4,5]. In addition to treatment, complexes hold great applications in the therapeutic and diagnostic of cancer. Other complexes containing zinc, gold, copper, nickel, titanium, rhodium, vanadium, cobalt, ruthenium and other metals are being given great attention as potential anticancer agents, due to their unique physiochemical properties [4,6,7].

The ability of complexes to adopt numerous three-dimensional structures allows them to have a diverse formation of functionalized targets of unique molecular structures, geometries and kinetic properties [4], which makes them promising candidates as anticancer agents. The geometry, oxidation and electronic state of the metal, due to partially filled *d*-orbitals, are the main factors in the activities and selectivities of the designed complexes. Another factor that also effects both selectivity and the activities of complexes is the existence of biologically relevant moieties as a counter ligand to the metal of interest.

One main strategy adopted in molecular design of the complexes is to change the central metal ion/atom/oxidation state that leads to change in the physiochemical, thermodynamic and kinetics properties, hence, making the complex scaffolds potential candidates for many pharmaceutical applications [8,9,10]. In addition, complexes can undergo ligand exchange reactions with proteins and enzymes.

Owing to their rapid access, availability and diversity, hydrazones are one of the most studied classes of compounds. Acylhydrazones have an additional donor site, namely C=O, which determines the versatility and flexibility of these compounds. Such molecules have attracted considerable attention for their antibacterial [11,12,13,14,15,16,17], antifungal [18,19] and antitumor activities [20,21,22]. Tagging the hydrazone moiety with another scaffold that can enhance the activity and/or physiological properties can advance the quest for suitable ligands.

In this contest, adamantane derivatives are known to exhibit considerable biological significance, which includes antiviral [23], antibacterial [24,25], anti-inflammatory [26,27] activities, and the inhibition of 11β-hydroxysteriod dehydrogenase type I [28]. A recent study discussed the uses of adamantine in resolution of racemic rimantadine hydrocholide [29]. In spite of biological importance of metal ions, only few Co(II), Cd(II) and Cu(II) complexes with hydrazone ligands incorporating adamantane were synthesized [30,31,32].

Quite recently, we had reported on the synthesis of new adamantane containing hydrazone compounds, where pyridine was one of the arms within the hydrazine-adamantyl compounds [33,34], where the nitrogen of the pyridyl group provides a supplementary position along imine one chelating different metallic ions [35]. Stimulated by our success in introducing the copper(II) complex of adamantne-1-carboxylic acid furan-2-ylmethylene hydrazide [36], and biologically active compounds [37,38,39], we planned to synthesize new complex compounds based on the adamantane-1-carboxylic acid pyridin-2-ylmethylene hydrazide (APH) and the metal ions Co(II), Ni(II), Cu(II) and Zn(II).

The selected metal ions in this study are of proven record as an essential element that plays a crucial rules in various biological settings [1,6,7]. The designed complexes were synthesized, characterized and thereafter screened against selected panels of cancer cell lines.

## 2. Results and Discussion

### 2.1. Synthesis and Characterization

**Scheme 1:**
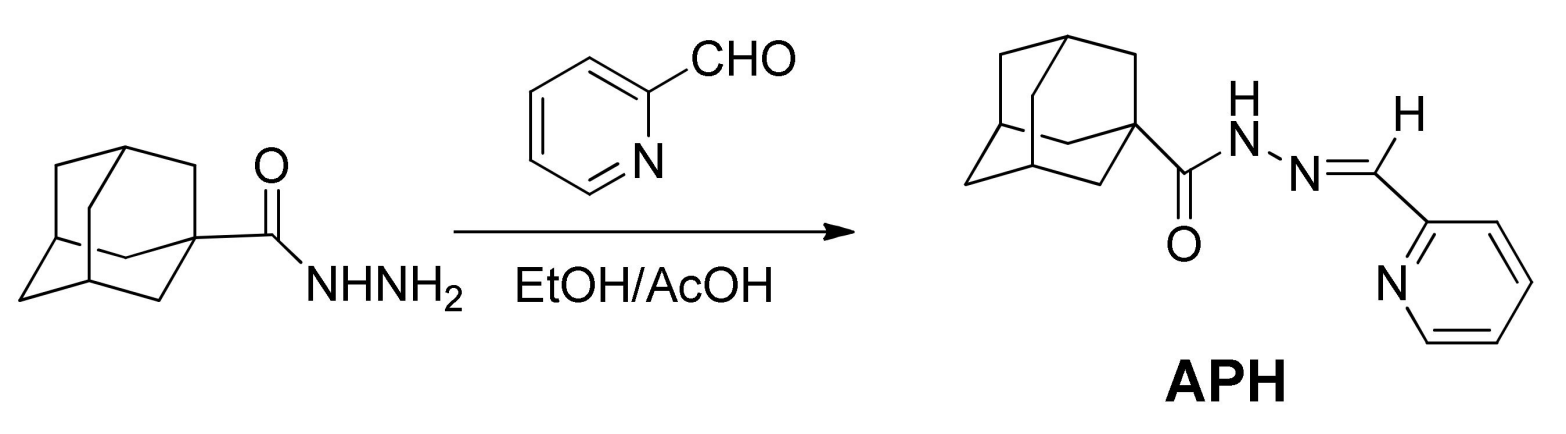
Synthesis of the ligand **APH**.

**Scheme 2:**
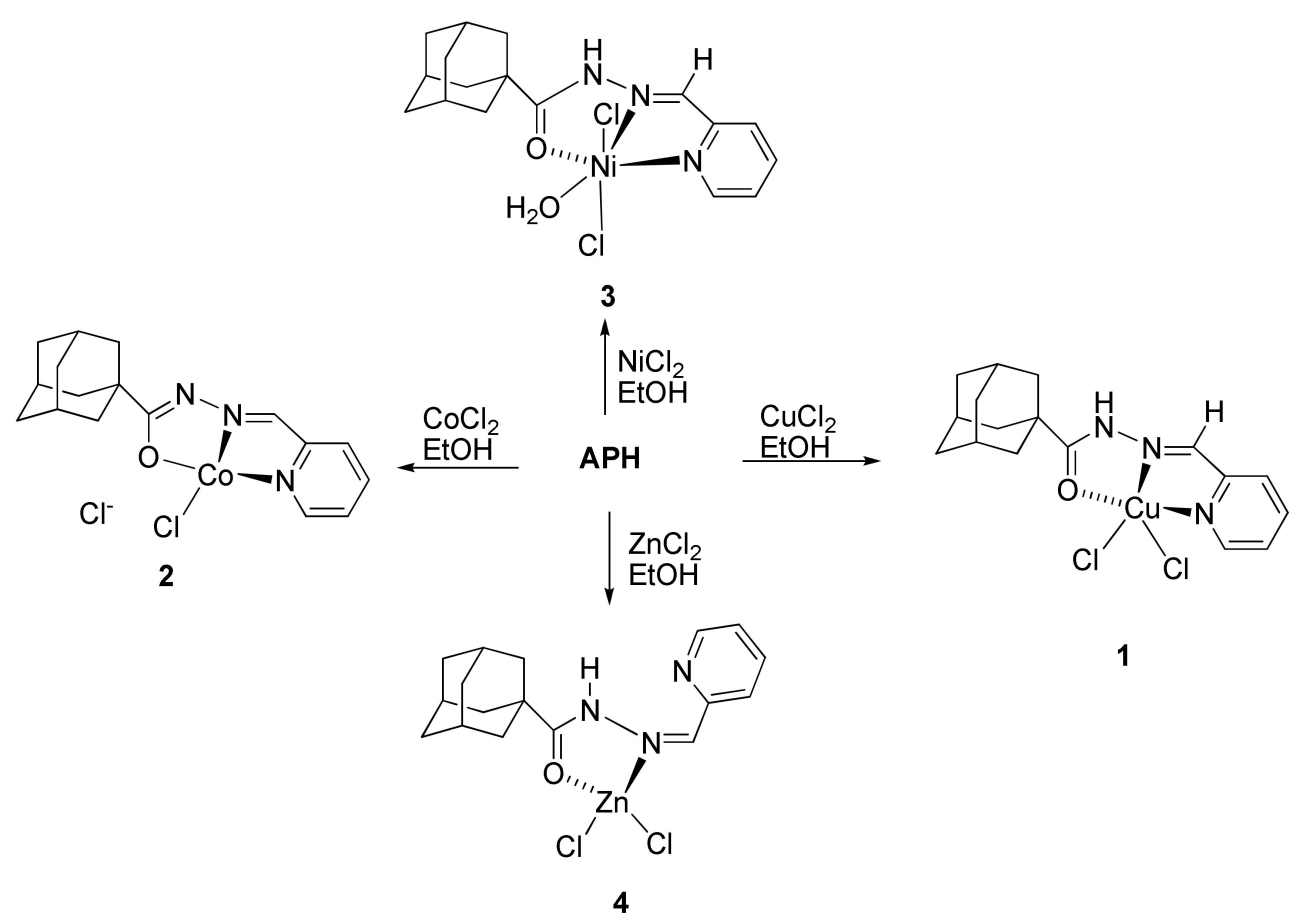
Synthesis of the complexes **1**–**4**. All reactions where performed under refluxing condition in ethanol.

The ligand Adamantane-1-carboxylic acid pyridin-2-ylmethylene hydrazide (**APH**) was obtained via condensation of 2-pyridinecarbaldehyde with adamantane-1-carboxylic acid hydrazide according to published procedures [33,34] as described in Scheme 1. All complexes obtained in good yield from the reaction of the metal chloride salts with ligand **APH** in refluxing ethanol for appropriate time, as shown in Scheme 2 All complexes were characterized using different spectroscopic techniques. High resolution mass spectra were obtained for all synthesized compounds, and the results confirmed the exact molecular formula and purity of the desired compounds. This mass spectrometric data supported the other data for the full assignments of the synthesized compounds.

### 2.2. FT-IR Spectra

IR spectra of complexes **1**, **2**, **3** and **4** analyzed in comparison to that of **APH**, recorded from 4000 to 400 cm^−1^. Table 1 shows the vibrations for selected groups within the ligand, as well as the complexes.

Compared with the ligand spectra **APH**, all complexes exhibit a shifted band from 1651 to 1588–1644 cm^−1^ assigned to ν(C=O). The calculated ν(C=O) is 1764 cm^−1^ where the hydrophobic interactions between adamantyl moieties are not included, as all calculations are performed on one **APH** unit. The ν(C=O) in the staggered conformer is lower in energy than the eclipsed conformation, due to it having less steric hindrance, as expected. The shift of the band to a lower wavenumber in the spectra of the complexes indicated the coordination of oxygen with metal ion, and the formation of M–O bond. The ν(C=N) band of the azomethine in the ligand at 1546 cm^−1^ is shifted to a lower wave number in the spectra of the complexes, 1523–1529 cm^−1^, suggesting its coordination with metal ion, and was also attributed to delocalization of the lone pair, and the formation of the Cu-N bond [40]. Low-energy in-plane and out-of-plane pyridine ring vibrations were shifted to higher frequencies in complexes **1**, **2** and **3** (from 670–684 cm^−1^), compared to that of **APH** (624–407 cm^−1^), which may be attributable to the rigidity of the system due to complexation [41,42,43,44]. All such observations were confirmed by the DFT calculations.

The absence of the carbonyl band in the spectra of complex **2** indicated that the hydrazone ligand had been coordinated to metal center through carbonyl oxygen after deprotonation. This evidence was strengthened via the presence of new stretching vibrations in the range 1090–1150 cm^−1^, attributed to the C–O in **2**. The ν(C–N) band at 640 cm^−1^ in **4** of the pyridine nitrogen in the complex is slightly shifted from that of the ν(C–N)py band (624 cm^−1^), indicating the absence of coordination of nitrogen with metal ion. The presence of a new band at about 3153 cm^−1^ was attributed to the ν(H–N), which indicated the formation of a hydrogen bond between the hydrogen atom of the NH and the nitrogen in the pyridine ring.

**Figure 1:**
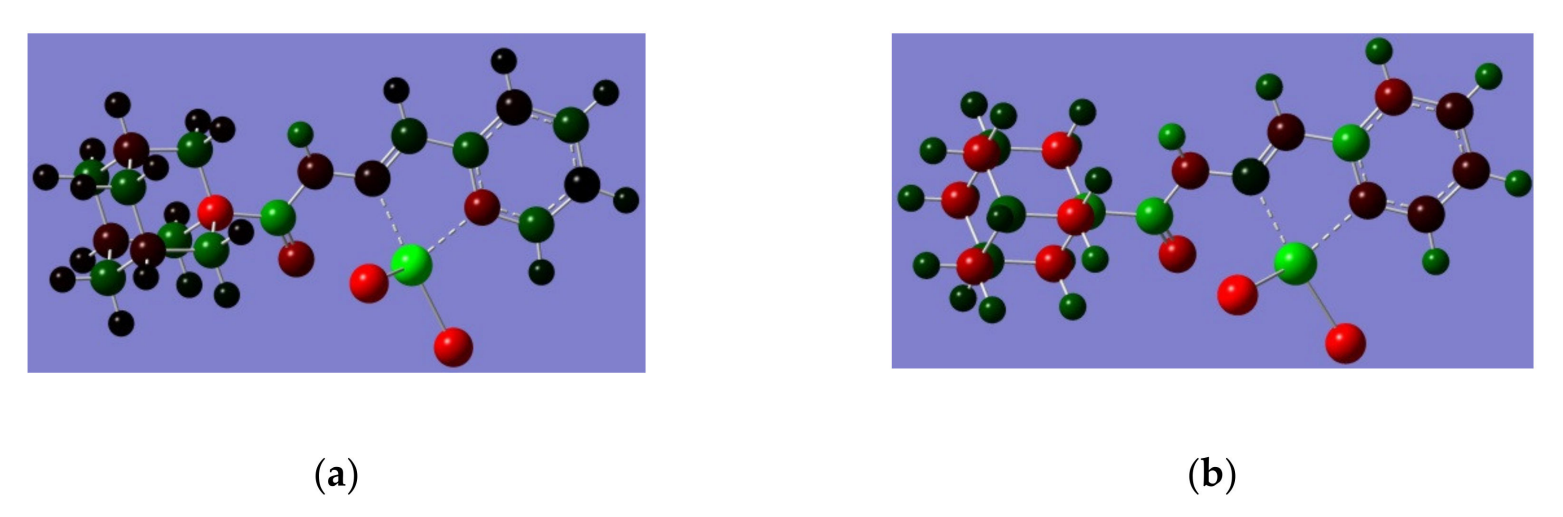
Electrostatic potential (ESP) for complex **1a** (**a**) and **1b** (**b**), where red presents the Mulliken negative charge distribution and green represents the positive charge distribution.

**Figure 2:**
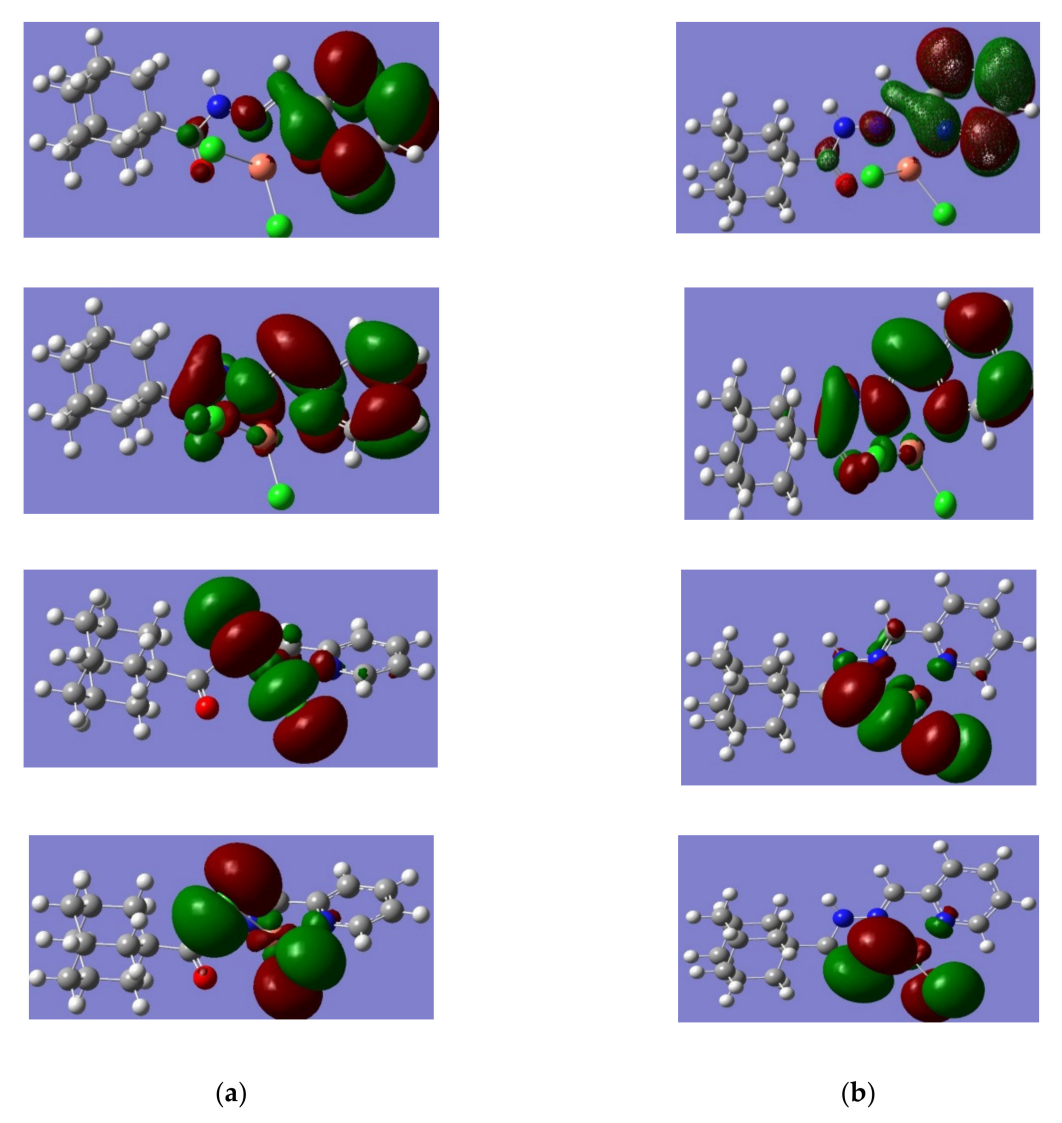
Presentation of the HOMO-LUMO atomic orbital for complex **1a** and complex **1b**, which are obtained at 6–311 + G(2d, p)/B3LYP level of theory (**a**) HOMO + 1, HOMO, LUMO, and LUMO − 1 for complex **1a** (**b**) HOMO + 1, HOMO, LUMO, and LUMO − 1 for complex **1b**.

The difference in the calculated corrected Gibbs free energy for complex **1** (eclipsed) for complex **1** (staggered) is −166 kcal/mole (Figure 1). Such lower energy of the eclipsed complex is due to the greater separation between the carbonyl oxygen from the chlorides, hence minimizing electron-electron repulsion. The HOMO-LUMO prsentation (Figure 2) shows relatively similar charge distributions, where the molecular orbital from the adamantyl group makes no contribution to similar molecular orbitals.

### 2.3. Magnetic Measurements and Molar Conductivities

The magnetic moment obtained at room temperature for complex **1** shows an μ_eff_ value of 1.94 B.M., corresponding to one unpaired electron and is indicative of a d^9^ system, which reveals the paramagnetic nature of the complex, indicating that copper in this complex is in the +2 oxidation state. The measured molar electrical conductivity of compound **1** is 277 Ω^−1^·cm^2^·mol^−1^ in water, indicating that the complex behaves as a 2:1 electrolyte. These results confirmed the lability of Cl^−^ anion in aqueous solution [45]. In the solid state, the Cl^−^ anion is bonded to the Cu(II) ion as confirmed by X-ray analysis described below.

Magnetic moments of the complex **2** shows μ_eff_ value of 4.64 B.M., which is indicative of a high spin d^6^ system, and reveals the paramagnetic nature of the complex; hence, cobalt, in this complex, is in the +3 oxidation state. The measured molar electrical conductivity of compound **2** in water is 121 Ω^−1^·cm^2^·mol^−1^, indicating that the complex behaves as a 1:1 electrolyte in aqueous solution.

The magnetic moment of complex **3** shows μ_eff_ value of 2.21 B.M., corresponding to two unpaired electrons, which is an indicative of d^8^ system, which reveals the paramagnetic nature of the complex; accordingly, nickel in this complex is in the +2 oxidation state. The measured molar electrical conductivity of compound **3** is 217 Ω^−1^·cm^2^·mol^−1^in water, indicating that the complex behaves as a 2:1 electrolyte. These results attested the lability of Cl^−^ anion in aqueous solution. In the solid-state, a chloride anion is coordinated to the Ni(II) ion.

The magnetic moment of the complex **4** is zero and the behavior is diamagnetic, as expected for d^10^ configuration. The measured molar electrical conductivity of compound **4** is 78 Ω^−1^·cm^2^·mol^−1^ in methanol, indicating that the complex behaves as a 1:1 electrolyte in methanolic solution: this result confirmed the lability of Cl^−^ anion in methanolic solution.

### 2.4. Structure Description of the Ligand and Complex **1**

#### 2.4.1. Crystal Structure of the Ligand APH

**Figure 3:**
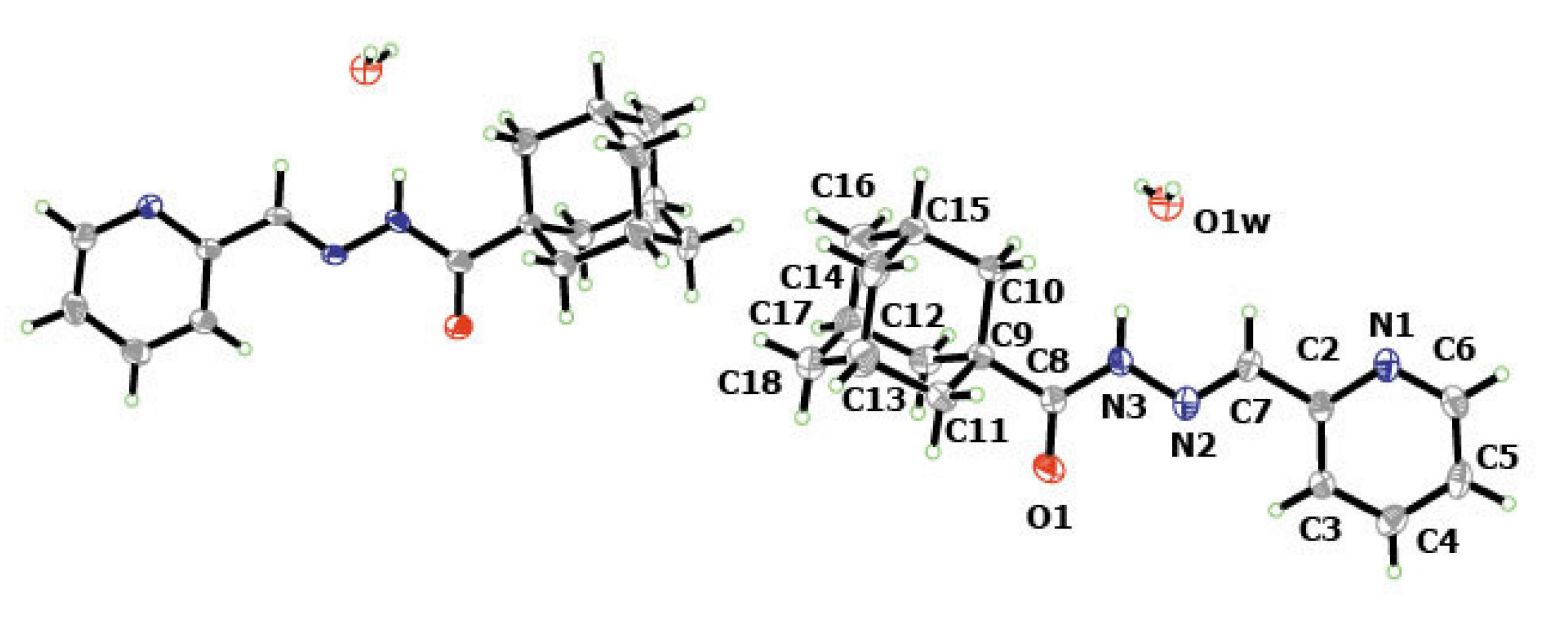
Molecular units of **APH** show two different crystallographic molecules in the unit cell. Ellipsoids are drawn at the 30% probability level. The numbered molecule is symmetrically equivalent to the other.

**Figure 4:**
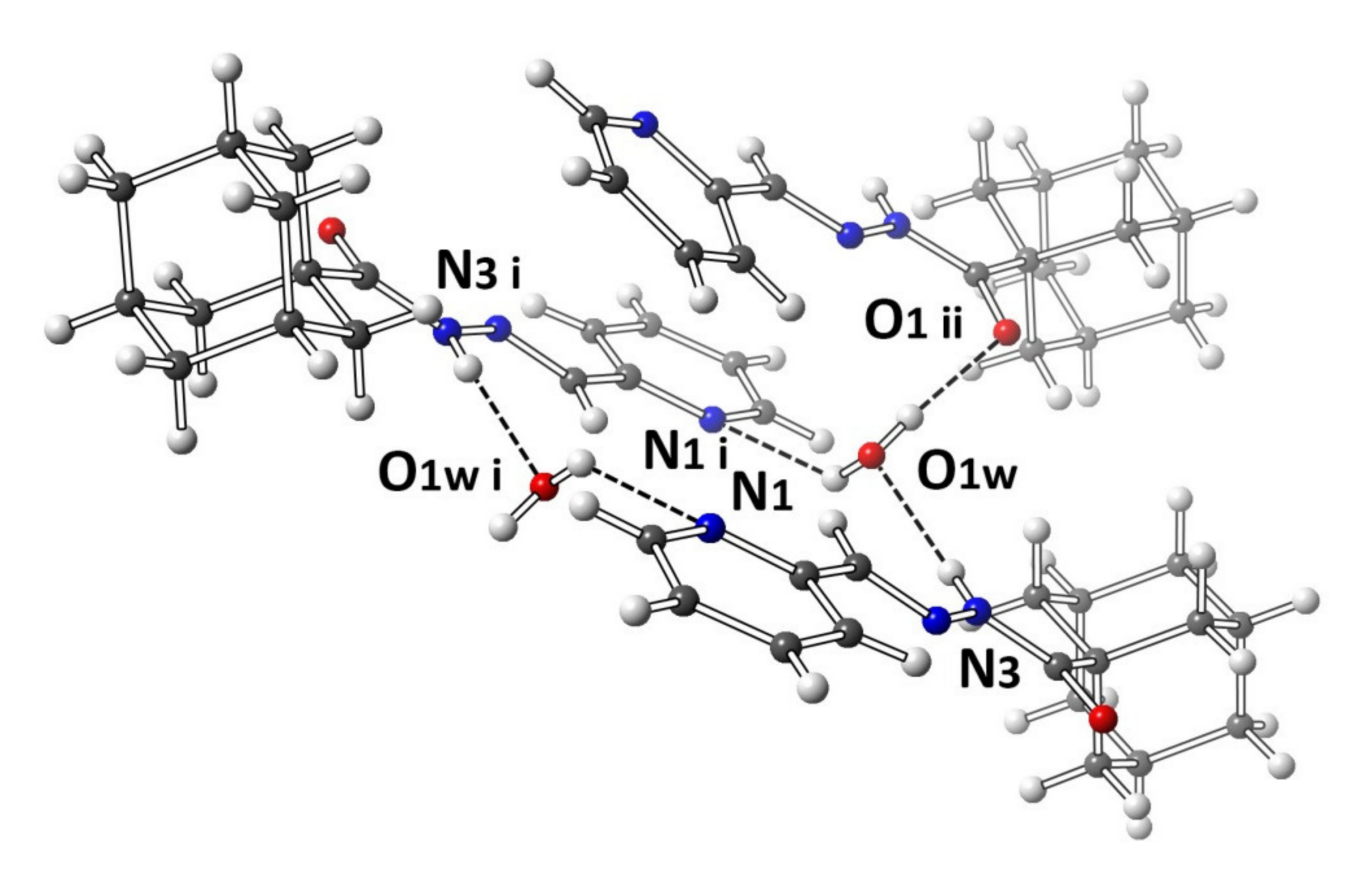
Molecular plot of **APH** shows the different intermolecular interactions between **APH** and water moieties. Only one asymmetric unit interaction is shown, as the other unit is symmetrically equivalent.

**Figure 5:**
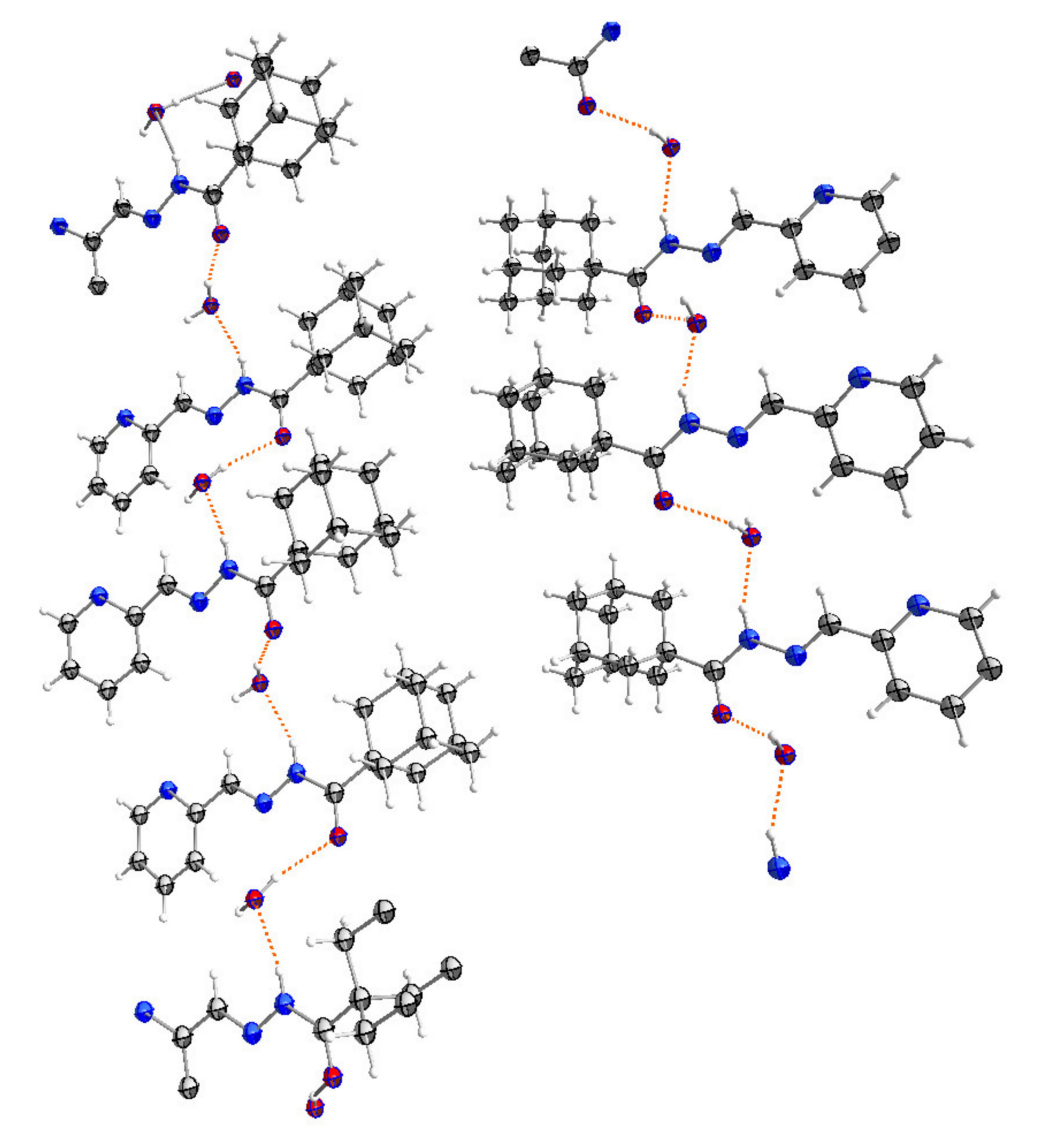
The packing diagram structures of **APH** with hydrogen bonds are shown as dotted lines.

A molecular structure view of **APH** with labeling and numbering of atoms, which is shown in Figure 3, along with selected crystalline structure parameters, is given in Table 2. The adamantyl groups constituting **APH** are disordered by 30° rotation (Figure 3), giving rise to two structures. The crystallized ligand with a molecule of water is essential for the H-bonding formation that is clearly connecting every other two ligands, as is shown in Figure 4. Figure 3 shows that the **APH**, in the free state, exists in the keto tautomeric form, and the structural conformation of the molecule is *E*, with respect to the imine double bond. The unit cell of the ligand (Figure 5) shows the head-to-head arrangements of the adamantyl moieties within the backed crystals, which can be attributed to the Van der Wall forces between the hydrophobic parts of the ligand units.

#### 2.4.2. Crystal Structure of **1**

**Figure 6:**
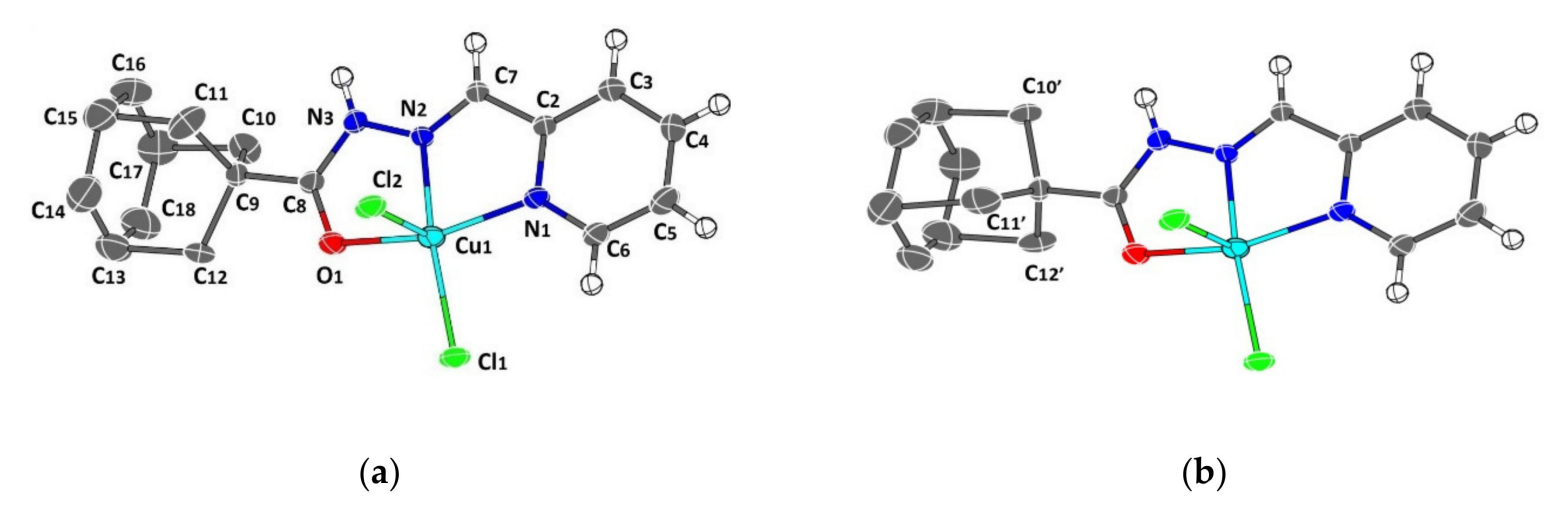
Thermal ellipsoid plot for the two possible disorder orientations in the adamantyl moiety (**a**) and (**b**) of **1**, drawn at the 30% probability level, with atomic labeling and numbering schemes. The crystalline solvent (CHCl_3_) and the hydrogen atoms on adamantyl have been omitted for clarity.

A crystal structure of **1** with an atom numbering scheme is shown in Figure 6. The bonding parameters are presented in Table 2. The crystal structure reveals that complex **1** crystallizes in a triclinic system, with a space group Pī. The unit cell of the crystal contains two molecules of (C_17_H_18_Cl_2_CuN_3_O). Complex **1** adopts a distorted square pyramidal structure. The Coordination sphere around copper(II) ion is satisfied with the five coordination environment, which is formed from the tridentate ligand; a pyridyl nitrogen atom (N_1_), an imino nitrogen (N_2_) and a keto oxygen atom (O_1_), along with two chloride ions.

In the complex, N_1_, N_2_, O_1_ and Cl_1_ occupy the basal plane of a distorted square pyramid, where the Cl_2_ is located in the apical position. The bond distances and bond angles of compound **1** are listed in Table 3.

**Figure 7:**
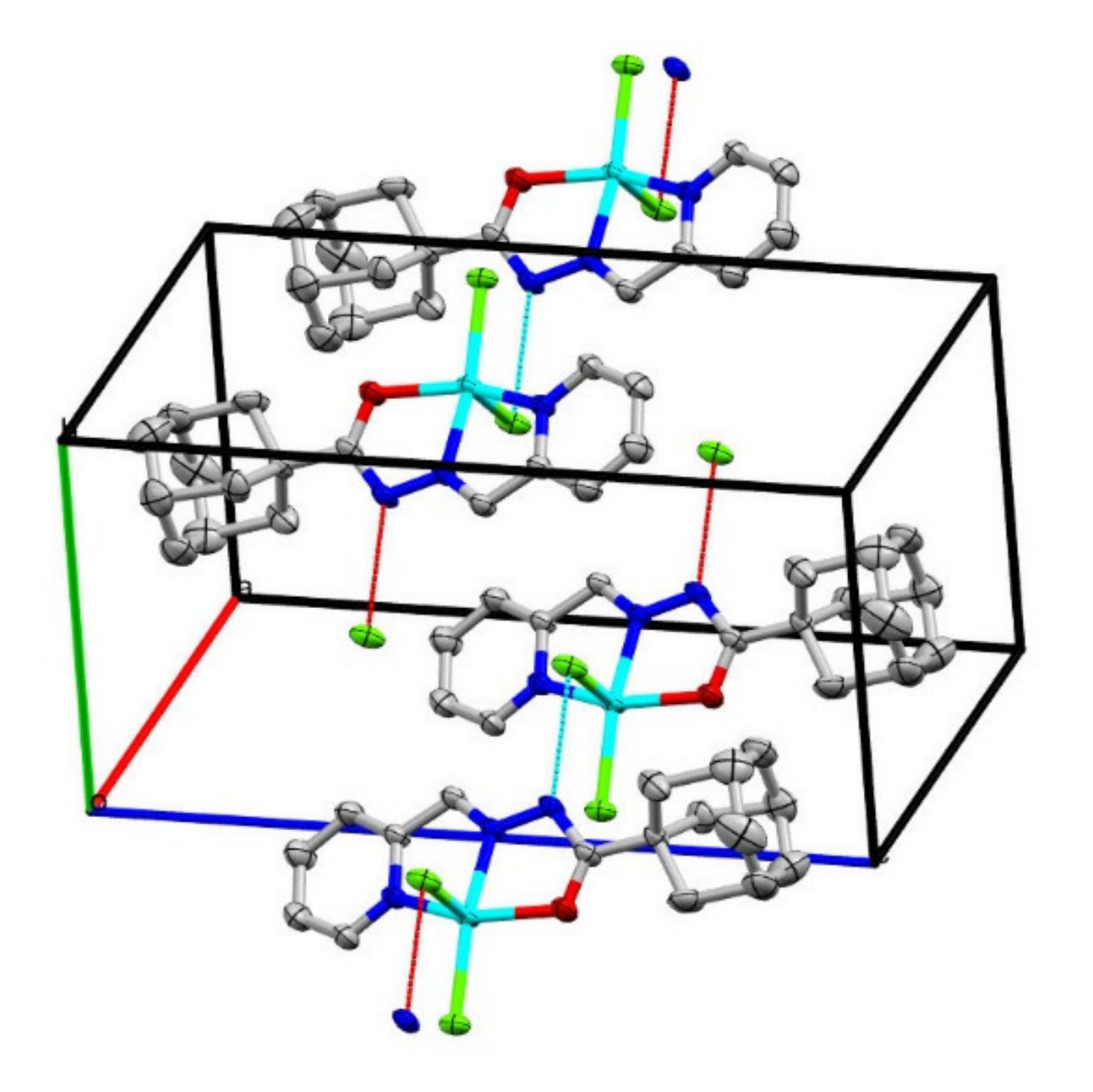
Packing diagram along c-axis showing the intermolecular interactions in **1**, the hydrogen atoms, disorder in adamantly and crystallization solvents were excluded for clarity purposes.

In the ligand **APH**, the C=O bond length is 1.229(6) Å, while in complex **1**, this bond is 1.234(7) Å, with uncertainty of the bond length within the experimental errors. Such difference in the bond lengths is attributed to the formation of the M–O bond and without the formation of an *enolate* bond with the metal. The presence of the hydrogen atom on N_3_ confirms the *keto* form of the coordinated ligand. The N_2_–Cu_1_–Cl_1_, N_2_–Cu_1_–Cl_2_, N_1_–Cu–N_2_, O_1_–Cu_1_–N_2_, N_1_–Cu_1_–Cl_1_ and O_1_–Cu_1_–Cl_1_ angles are 161.85(14)°, 96.26(14)°, 78.52(16)°, 77.12(16)°, 98.58(13)° and 99.42(11)°, respectively. The bond distances and angles are comparable to those observed for similar kinds of complexes [45]. A molecule of chloroform as a solvent of crystallization is observed within the crystal of our complex. The only intermolecular force that can be considered is N–H…Cl, with a distance of [3.150 Å between Cl_2_…N_3_], as shown in Figure 7.

Applying continuous shape measures (CShM) on Cu(II) ion by comparing with all reference standard 5-coordinate polyhedrons were calculated using SHAPE 2.0 program [46]. This five-coordination sphere and geometry can be best described as a square pyramid (*C*_4v_) with CShM = 1.545, in comparison with the closest value for a vacant octahedron (a regular polyhedron with one or two vertices removed) with CShM = 3.186.

### 2.5. Cytotoxicity Assay

Adamantane derivatives are found to have numerous biological activities, angiogenesis inhibition [47], antiviral [48,49,50], anti-inflammation [17], antimicrobial [50,51,52,53,54] and anticancer [54,55,56,57]. Adamantyl hydrazone complexes with metals had been heavily studied during the past few years for their antitumor activity against many cancer cell lines [58,59].

In this work, the cytotoxicity of four different complexes Cu(II) (**1**), Co(II) (**2**), Ni(II) (**3**) and Zn(II) (**4**) containing adamantne-1-carboxylic acid pyridin-2-ylmethylene hydrazide **APH** are studied.

Six different cancer cell lines (MCF7, T47D, MDA-MB-23, PC3, DU145 and Caco-2) are used to assess the effects of those compounds on cell proliferation. Two different concentrations of the test compounds are used: 12.5 μM and 50 μM. The % of cell growth inhibition is assessed by an MTT assay. The obtained results are compared with the ligand **APH**. In addition, doxorubicin (a well-known antitumor drug that is used in the treatment of several types of cancers by activating apoptotic pathways, leading to the death of these cells [60]), is used as a positive control.

As shown in Table 4, all of the tested compounds have shown an antiproliferative activity against the used cell lines when they are compared with the parent ligand **APH** at both treatment doses of 12.5 and 50 µM. Compounds **1** and **2** are the most cytotoxic of them all. Compound **1** has shown activities of 93.30 ± 1.20%, 88.60 ± 1.17%, 90.26 ± 0.55%, 89.88 ± 0.39%, 89.85 ± 0.73%, 87.06 ± 1.57% of cell viability inhibition in MCF7, T47D, MDA-MB-23, PC3, DU145 and Caco-2 cell lines, respectively. Compound **2**, with a concentration of 50 µM (Table 4) has shown cytotoxic activities of 89.35 ± 1.22%, 86.03 ± 0.99%, 84.52 ± 2.54%, 88.60 ± 0.44%, 87.78 ± 1.32%, 85.22 ± 0.65% in MCF7, T47D, MDA-MB-23, PC3, DU145 and Caco-2 cell lines, respectively. Compounds **3** and **4** are also found to be toxic to cells, but with lower impacts on cell viability inhibition than compounds **1** and **2**, with a % of growth inhibition of 37.29 ± 2.71 for MCF7, 44.78 ± 2.10 for MDA-MB-231, 62.47 ± 0.14 for PC3, and 64.28 ± 2.78 for CaCo2 for compound **3**. Compound **4** of 50 µM concentration has cytotoxic activities of 64.83 ± 1.18 for MCF7, 32.31 ± 1.95 for MDA-MB-231, 84.93 ± 0.53 for PC3, 71.62 ± 1.46 for DU145 and 60.32 ± 1.43 for the Caco-2 cell line. Other studies have reported that copper (II) complexes with 1-adamantoyl hydrazone bearing pyridine rings are found to induce apoptosis and inhibit agiogenesis in some tumor cell lines [61].

There was no significant difference in the % of cell viability inhibition values between the different cell lines used in this study, following the treatment with compounds **1** and **2**. On the other hand, compounds **3** showed a stronger antiproliferative effect against the two prostate cancer cell lines and Caco-2 (the colon cancer cell line), compared to their effect on the three breast cancer cell lines. Compound **4** caused more cell viability inhibition in the two prostate cancer cell lines than the other ones.

Doxorubicin, an anthracycline antibiotic and one of the most effective anticancer drugs against breast cancer since 1970 [62], showed an inhibition of cell viability ranging from 35.41% to 65.68%, and from 41.06% to 72.23% at 12.5 µM and 50 µM treatment doses, respectively. At a concentration of 12 µM, doxorubicin was showing stronger cytotoxic effects on cells, compared with our four test compounds, except for the CaCo-2 cell line, where compound **1** was found to be more cytotoxic to these cells with a 2-fold increase in the % of viability inhibition than doxorubicin (Table 4). At 50 µM treatment dose, compounds **1** and **2** showed a stronger antiproliferative activity on the three breast cancer cell lines used in this study than doxorubicin; compounds **1**, **2**, and **3** were more cytotoxic to PC3 cells compared to doxorubicin; and all four tested compounds were more cytotoxic to DU145 and CaCo-2 cells when compared with doxorubicin (Table 4).

It is concluded that the cytotoxicity effect of **APH** against different tumor cell lines has been improved through its complexation with different metals, hence, making these complexes possible candidates as anticancer drugs. Furthermore, future studies shall be conducted on these newly synthesized bioactive compounds, in order to understand the mechanisms of the cytotoxic and mutagenic changes they may cause cancer cells.

## 3. Materials and Methods

### 3.1. General Methods

Starting materials and solvents used were analytical grades and provided from Scharlau, Fluka and Sigma Aldrich (St. Louis, MO, USA). All reactions were monitored by thin layer chromatography (TLC) (Merck, Darmstadt, Germany) using Merck aluminum plates, which are pre-coated with silica gel PF254. The ^1^H− and ^13^C-NMR spectra were recorded on 300 MHz Bruker AVANCE (Bruker BioSpin, Ettlingen, Germany). Chemical shifts were reported as δ values in ppm. Spectra were acquired in either DMSO-d_6_ or CDCl_3_ (Scharlau, Fluka and Sigma Aldrich (St. Louis, MO, USA). High-resolution mass spectra (HRMS) were measured (in positive/or negative ion mode) using the electrospray ion trap (ESI) technique by collision-induced dissociation on a Bruker APEX-IV (7 Tesla), (Bruker Daltonics, Bremen, Germany) instrument. Melting points (mp) were determined on an Electrothermal Melting point Apparatus, and were reported as acquired without corrections. The infrared spectra were recorded from 4000 to 400 cm^−1^ region with a Nicolet Impact-400 FT-IR spectrophotometer (Thermo Fisher Scientific, Waltham, MA, USA) using KBr pellets.

### 3.2. Syntheses

#### 3.2.1. Synthesis of the Hydrazone Ligand (**APH**)

The hydrazone ligand was prepared by adding 2-pyridinecarbaldehyde (1.1 mmol) to a solution of 1 mmol of adamantane-1-carboxylic acid hydrazide in absolute ethanol containing six drops of glacial acetic acid [33,34]. The mixture was refluxed for 4 h, and the reaction progress was monitored by TLC. The mixture was then poured into cold water and the formed precipitate was filtered, washed with petroleum ether, and then recrystallized from ethanol to produce white crystals (90% Yield, mp 136–137 °C).

^1^H-NMR (DMSO-d_6_, 300 MHz, in ppm): δ 1.73 (s, 6H, Adamantane-H); 1.81 (s, 6H, Adamantane-H); 1.89 (s, 3H, Adamnatane-H); 7.34–8.52 (m, 4H, H-pyridine); 8.34 (s, 1H, HC=N); 11.06 (br s, 1H, O=C–NH). ^13^C-NMR (DMSO-d_6_, 75 MHz, in ppm): δ 27.5 (3C, Adamantane-C); 38.4 (3C, Adamantane-C); 36.1 (3C, Adamantane-C); 146.9 (C=N); 173.9 (C=O); 120.0–149.6 (4C, C-pyridine); 153.6 (C–N)py. HRMS (ESI, positive ion mode): *m*/*z* 284.17544 [(M + H)^+^ Calcd for C_17_H_22_N_3_O: 284.17574]; Anal. Calcd for C_17_H_21_N_3_O·H_2_O: C, 67.87; H, 7.84; N, 14.12; O, 10.17. Found: C, 67.75; H, 7.69; N, 13.94; O, 10.62%.

#### 3.2.2. Preparation of the Complexes

##### General Procedure for the Preparation of the Complexes

The complexes were prepared by adding equimolar amounts of the appropriate metal (II) chloride hydrate to a magnetically stirred ethanol solution of **APH**. The resultant solution was refluxed for 2–5 h. The produced solid complexes were filtered off, washed with cold ethanol, dried under vacuum and recrystallized from an appropriate solvent.

##### Synthesis of **1** (C_17_H_21_Cl_2_CuN_3_O)

Recrystallized from CHCl_3_. Green crystals (0.350 g, 70% yield, mp 245 °C (decomposition)). Selected FTIR (KBr): $\overset{¯}{v}$_max_ (cm^−1^) = (C=O) 1588 s, (C=N) 1527 s; (C–N)py 684 m. HRMS (ESI, positive ion mode): *m*/*z* 381.06681 [(M–Cl)^+^; Calcd for C_17_H_21_ClCuN_3_O: 381.06637]; Anal. Calcd for C_17_H_21_Cl_2_CuN_3_O: C, 48.87; H, 5.07; N, 10.06. Found: C, 48.95; H, 5.12; N, 10.13.

##### Synthesis of **2** (C_17_H_21_Cl_2_CoN_3_O)

Recrystallized from CHCl_3_. Green crystals (0.260 g, Yield: 52%, mp 300 °C (decomposition).

Selected FTIR (KBr): $\overset{¯}{v}$_max_ (cm^−1^) = (C=N) 1615 s, (C=N) 1529 s; (C–O) 1146 s; (C–N)py 677 m. HRMS (ESI, positive ion mode): *m*/*z* 377.06950 [[M–Cl]^+^, Calcd for C_17_H_21_ClCoN_3_O: 377.06997]; Anal. Calcd for C_17_H_21_Cl_2_CoN_3_O: C, 49.53; H, 4.89; N, 10.19. Found: C, 49.75; H, 4.93; N, 10.25%.

##### Synthesis of **3** (C_17_H_21_Cl_2_N_3_NiO)

Green powder (0.290 g, 58% yield, mp 300 °C (decomposition)).

Selected FTIR (KBr): $\overset{¯}{v}$_max_ (cm^−1^) = (C=O) 1606 s, (C=N) 1523 s; (C–N) py 670 m. HRMS (ESI, negative ion mode): *m/z* 376.07218 [(M–H_2_O–Cl)^−^; Calcd for C_17_H_21_ClN_3_NiO: 376.07211].

##### Synthesis of **4** (C_17_H_21_Cl_2_N_3_OZn)

White powder (0.350 g, 70% yield, mp 300 °C (decomposition)).

^1^H NMR (DMSO-d6, 300 MHz, in ppm): δ 1.73 (s, 6H, Adamantane-H); 1.81 (s, 6H, Adamantane-H); 1.89 (s, 3H, Adamnatane-H); 7.40 d; 7.87 m (2H); 8.39 s (N=CH); 8.54 d (1H); 11.36 s (1H, NH). ^13^C NMR (DMSO-d6, 75 MHz, in ppm): δ 26.1 (3C, Adamantane-C); 27.6 (3C, Adamantane-C); 38.3 (3C, Adamantane-C); 146.3 (C=N); 120.9, 124.8, 137.5, 149.5, 153.0 (C-pyridine); 174.4 (C=O). Selected FTIR (KBr): $\overset{¯}{v}$_max_ (cm^−1^) = (C=O) 1615 s, (C=N) 1529 s; (C–N) py 677 m. HRMS (ESI, negative ion mode): *m*/*z* 416.02835 [(M–H)^−^; Calcd forC_17_H_20_Cl_2_ZnN_3_O: 416.02801]; Anal. Calcd for C_17_H_21_Cl_2_N_3_OZn: C, 48.65; H, 5.04; N, 10.01. Found: C, 48.45; H, 5.12; N, 10.09.

### 3.3. X-Ray Crystallography

Single crystal X-ray diffraction data were measured using Oxford diffraction Xcalibur (Mo) X-ray source (λ = 0.71073 Å) at 293(2) K. Data collection, reduction and cell refinement were performed using CrysAlisPro [63]. The structure solutions and refinements were carried out using Olex2 [64] and the implemented SHELXT and SHELXS programs [65]. All non-hydrogen atoms were refined anisotropically. Hydrogens were located in a difference Fourier map and refined isotropically. All hydrogens were positioned geometrically and set to ride on their respective parent. The molecular graphics and crystallographic illustrations for **APH** and complex **1** were prepared using relevant crystallographic data. The structure refinement parameters for **APH** and complex **1** are summarized in Table 2. Selected bond lengths and angles for **APH** and **1** are listed in Table 3 and Table 5, respectively. Important intermolecular interactions for **APH** are listed in Table 6. CCDC-1558282 (for **APH**) and CCDC-1558283 (for **1**) contain the Supplementary Materials crystallographic data this article. These data can be obtained free of charge at http://www.ccdc.cam.ac.uk/conts/retrieving.html, or from the the Cambridge Crystallographic Data Center, 12 Union Road, Cambridge CB2 1EZ, UK; Fax: +44-1223-336-033; or by e-mail: deposit@ccdc.cam.ac.uk.

### 3.4. Computational Methods

Structures of all complexes were optimized in silico gas phase using DFT methods with the Pople valence triple zeta basis set with added diffuse and polarization functions 6–311 + G (2d, p). No negative or imaginary frequencies were obtained for any structures. All calculations were performed on G16 running HPC cluster. Calculations were on conducted on distorted square pyramidal on complex **1**, seesaw geometry on complex **2**, distorted square pyramidal on complex **3** and distorted tetrahedral geometry on complex **4**. The optimized structure of complex **1** is in complete agreement with the obtained single crystal structure one. In order to validate the calculations on molecules using the previously mentioned methods, another DFT calculation was conducted on complex **1** with Ahlrich valence triple zeta basis set with added polarization functions DefTZVP. Results from both basis sets eluded the same frequencies as assigned in Table 3. The discrepancies between the obtained experimental frequencies with the calculated ones can be attributed to the solvent molecules that are trapped in the crystals.

### 3.5. Cytotoxicity Assay

#### 3.5.1. Cell Lines

Three selected human breast cancer cell lines were used in this study namely: the Michigan Cancer Foundation-7 (MCF7), human breast cancer cell line, commonly used in biomedical research, involving the hormonal expression of cancer cells (T-47D), and M.D. Anderson and MB Metastasis Breast cancer (MDA-MB-231). Two human prostate cancer cell lines: the prostate cancer 3 cell line (PC3) the human androgen-independent prostate cancer cell line (DU145); and one human colorectal cancer cell line (Caco-2).

#### 3.5.2. Cell Culture

The MCF7, T47D, MDA-MB-231, DU145 and PC3 cell lines were cultured using RPMI 1640 medium, whereas DEMEM medium was used for the Caco-2 cell line. The media were supplemented with 10% fetal bovine serum (FBS) (Gibco Invitrogen Corporation, Grand Island, NY, USA), 1% of 2 mM L-glutamine, 50 IU/mL penicillin and 50 μg/mL streptomycin (Lonza, Verviers, Belgium). All cells were grown at 37 °C in a 5% CO_2_ incubator, and were harvested using trypsin-EDTA solution.

#### 3.5.3. Viability Assay

Cells from each cell line were seeded at a density of 10 × 10^3^ cells/well in 96-well plates and allowed to attach overnight. Cells were treated with two different concentrations (12.5 μM and 50 μM) of the tested compounds **1**, **2**, **3**, **4** and doxorubicin, which was used as a positive control. The experiment was done in 4 replicas. After 72 h treatment, MTT (3-(4,5-dimethylthiazol-2-yl)-2,5-diphenyl-tetrazolium bromide) assay was performed according to the cell proliferation assay kit (Promega, Madison, WI, USA). Absorbance (OD) was measured at 570 nm, with background subtraction at 630 nm. DMSO was used as a negative control, and the percentage of viability inhibition was measured as an indication of the cytotoxicity of the tested compounds.

## 4. Conclusions

In the present work, we have reported the synthesis, characterization and anticancer activities of four new adamantylpyridylhydrazone complexes. All prepared complexes contain the new tridentate N,N,O-donor Schiff base, which is derived from theadamantylpyridylhydrazone nucleus. One crystalline complex (**1**) was analyzed using its X-ray single structure analysis, and it was found that it exists in a square pyramidal geometry. Complex **2** exists in the form of Co(III) ions. Further investigations of the structure activity-relationship studies of our new complexes are in progress in our laboratory, and the results will be reported in due course.

## Figures and Tables

**Table 1:** Selected vibrational bands (cm^−1^) of the ligand and its complexes.

Compound	υ (C=O)	υ (C=N)	υ (C=N)PY
APH	1651 (s)	1546 (s)	624 (m)
	1764 *	1600 *	636 *
	1757 *#	1601 *#	633 *#
1	1588 (s)	1615, 1527 (s)	684 (m)
	1757 (calc′d) *	1657, 1600 (calc′d) *	761 (calc′d) *
	1766 (calc′d) **	1664, 1605 (calc′d)	767 (calc′d)
2	1146 (s) υ(C–O)_coor_	1515 (s)	677 (m)
	1145 *	1512 *	683 *
3	1608 (s)	1523 (s)	670 (m)
4	1644 (s)	1529 (s)	640 (m)
	1666 *	1532 *	647 *

* DFT calculated bands with basis set 6–311 + G(2d, 1p); ** DFT calculated bands with basis DefTZVP; # C=O group is staggered with adamantyl moieties.

**Table 2:** Crystallographic and refinement data for **APH** and **1**.

	APH	1
Chemical formula	C_17_H_23_N_3_O_2_	C_18_H_19_C_l5_CuN_3_O
Formula weight	301.38	534.15
Crystal system	monoclinic	Triclinic
Space group	P2_1_/c	Pī
Z	8	2
a (Å)	38.328(3)	6.4038(13)
b (Å)	6.9156(5)	9.676(3)
c (Å)	11.9183(12)	18.706(4)
α(°)	90	76.86(2)
β (°)	95.099(10)	86.369(17)
γ (°)	90	81.46(2)
V (Å^3^)	3146.6(5)	1115.7(5)
Density (Mg/m^3^)	1.272	1.590
Absorption coefficient (mm^−1^)	0.085	1.592
F(000)	1296.0	540.0
2Ѳ Range for data collection (°)	5.89–50.05	6.44–50.1
Reflections collected	13,020	6883
Independent reflections (R_int_)	5557 (0.0673)	3948 (0.0352)
Goodness-of-fit, F^2^	1.052	1.035
R indices (all data)	R_1_ = 0.1943; wR_2_ = 0.2330	R_1_ = 0.0878; wR_2_ = 0.1373
Final R indices [I > 2σ(I)]	R_1_ = 0.0893; wR_2_ = 0.1733	R_1_ = 0.0584; wR_2_ = 0.13173
Largest difference in peak and hole (e Å^−3^)	0.23 and −0.25	0.60 and −0.65
$R_{1}=\sum \left|F_{0}\right|-\left|F_{c}\right|/\sum \left|F_{0}\right|$, $wR_{2}={\left\{\sum w{\left(F_{0}^{2}-F_{c}^{2}\right)}^{2}/\sum {\left[wF_{0}^{2}\right]}^{2}\right\}}^{1/2}$		

**Table 3:** Selected bond lengths (Å) and angles [°] for **1**.

Bond Distances			
Cu_1_–Cl_2_	2.460(2) 2.231 * 2.228 **	Cu_1_–Cl_1_	2.212(2) 2.198 * 2.198 **
Cu_1_–N_2_	1.967(4) 2.136 * 2.159 **	Cu_1_–O_1_	2.065(4) 2.948 * 2.955 **
Cu_1_–N_1_	2.026(5) 2.122 * 2.131 **	N_3_–N_2_	1.364(6) 1.348 * 1.341 **
N_3_–C_8_	1.346(7) 1.399 * 1.398 **	N_1_–C_6_	1.328(7) 1.329 * 1.328 **
N_1_–C_2_	1.349(6) 1.348 * 1.346 **	C_8_–O_1_	1.239(6) 1.210 * 1.207 **
**Angles**			
Cl_1_–Cu_1_–Cl_2_	101.79(6) 106.34 * 106.69 **	N_2_–Cu_1_–Cl_2_	96.26(14) 92.46 * 92.47 **
N_2_–Cu_1_–Cl_1_	161.85(14) 158.44 * 158.26 **	N_2_–Cu_1_–O_1_	77.12(16) 60.53 * 60.32 **
N_2_–Cu_1_–N_1_	78.52(16) 76.32 * 75.79 **	O_1_–Cu_1_–Cl_2_	94.45(13) 83.88 * 83.19 **
O_1_–Cu_1_–Cl_1_	99.42(11) 110.23 * 110.95 **	N_1_–Cu_1_–Cl_2_	104.39(14) 132.76 * 133.24 **
N_1_–Cu_1_–Cl_1_	98.58(13) 97.46 * 97.30 **	N_1_–Cu_1_–O_1_	150.58(16) 125.15 * 124.71 **

* calculated on basis set used 6–311 + G(2d, 1p); ** calculated on basis used DefTZVP.

**Table 4:** The effect of newly synthesized complexes **1**, **2**, **3** and **4**, derived from adamantyl containing a hydrazine (**APH**) ligand on the viability of six cancer cell lines (MCF7, T47D, MDA-MB-231, PC3, DU145, and Caco-2) after 72 h of incubation. Doxorubicin is used as a positive control. The data are represented as percentage of growth inhibition ± SD.

Compound No	Concentration (µM)	MCF7	T47D	MDA-MB-231	PC3	DU145	Caco-2
**APH**	12.5	2.66 ± 0.20	5.39 ± 0.46	5.90 ± 2.12	15.82 ± 6.67	24.15 ± 0.94	17.17 ± 1.50
	50	13.68 ± 1.87	21.98 ± 3.66	22.76 ± 3.01	19.99 ± 0.53	46.39 ± 2.78	43.62 ± 1.39
**1**	12.5	15.33 ± 0.58	28.32 ± 1.40	32.24 ± 3.84	56.74 ± 4.52	25.30 ± 3.96	65.06 ± 1.37
	50	93.30 ± 1.20	88.60 ± 1.17	88.60 ± 1.17	89.88 ± 0.39	89.85 ± 0.73	87.06 ± 1.57
**2**	12.5	33.34 ± 4.60	22.64 ± 5.04	30.78 ± 1.87	40.67 ± 1.32	49.19 ± 2.98	74.89 ± 1.58
	50	89.35 ± 1.22	86.03 ± 0.99	84.52 ± 2.54	88.60 ± 0.44	87.78 ± 1.32	85.22 ± 0.65
**3**	12.5	17.32 ± 1.63	11.33 ± 1.84	18.43 ± 2.01	32.78 ± 2.63	30.28 ± 3.93	15.90 ± 1.87
	50	37.29 ± 2.71	28.99 ± 4.34	44.78 ± 2.10	62.47 ± 0.14	61.71 ± 2.09	64.28 ± 2.78
**4**	12.5	6.86 ± 3.40	7.21 ± 3.66	8.62 ± 1.49	10.76 ± 1.02	28.87 ± 3.87	20.39 ± 5.74
	50	64.83 ± 1.18	25.26 ± 7.97	32.31 ± 1.95	84.93 ± 0.53	71.62 ± 1.46	60.32 ± 1.43
Doxorubicin	12.5	63.43 ± 1.34	47.81 ± 4.18	65.68 ± 0.12	63.99 ± 0.46	53.53 ± 1.41	35.41 ± 2.16
	50	72.23 ± 1.08	54.58 ± 2.23	71.52 ± 1.18	67.61 ± 0.47	52.12 ± 2.12	41.06 ± 3.21

**Table 5:** Selected bond lengths (Å) and angles (°) for ligand **APH.**

Bond Distances			
C_2_–C_7_	1.462(7)	C_2_–N_1_	1.339(6)
C_2_–C_3_	1.401(6)	N_2_–C_7_	1.271(6)
N_2_–N_3_	1.384(5)	N_3A_–C_8A_	1.358(6)
N_3_–C_8_	1.338(6)	C_2A_–C_3A_	1.362(7)
N_1_–C_6_	1.341(6)	C_8A_–O_1A_	1.215(5)
C_8_–C_9_	1.521(7)	N_3A_–N_2A_	1.363(5)
C_8_–O_1_	1.229(6)	N_2A_–N_3A_	1.362(6)
Angles			
C_8_–N_3_–N_2_	117.6(4)	O_1_–C_8_–N_3_	122.3(5)
C_7_–N_2_–N_3_	116.6(5)	O_1_–C_8_–C_9_	119.4(5)
N_1_–C_2_–C_7_	115.0(5)	O_1A_–C_8A_–C_9A_	121.1(5)
O_1A_–C_8A_–N_3A_	121.4(5)	C_7A_–N_2A_–N_3A_	118.0(4)

**Table 6:** Some important intermolecular interactions in **APH**.

Donor-H…Acceptor		D-H	H…A	D…A	D-H…A
N_3A_-H_3A_…O_2W_	x,1/2 − y,1/2 + z	0.86	2.07	2.8697(3)	155
N_3_-H_3_…O_1W_	x, 3/2 − y,1/2 + z	0.86	2.05	2.8557(3)	156
O_1W_-H_1WA_…N_1_	x, 3/2 − y,1/2 + z	0.85	2.06	2.8669(3)	158
O_2W_-H_2WA_…N_1A_	x,1/2 + y,1/2 − z	0.70	2.38(6)	2.8799(3)	156
O_2W_-H_2WB_…O_1A_		0.85	2.07(7)	2.8943(3)	124
O_1W_-H_1WB_…O_1_		0.85	2.07(6)	2.9175(8)	176

## Supplementary Materials

The Supplementary Materials are available online.
