# Supplementary material for: Synthesis, Characterization and Biological Evaluation of Metal Adamantyl 2-Pyridylhydrazone Complexes
Source: Molecules. 2020 May 29;25(11):2530. doi: 10.3390/molecules25112530 (PMC7321243; doi:10.3390/molecules25112530)
Supplement: Supplementary file 1 [file molecules-25-02530-s001.zip › Spectra.pdf]

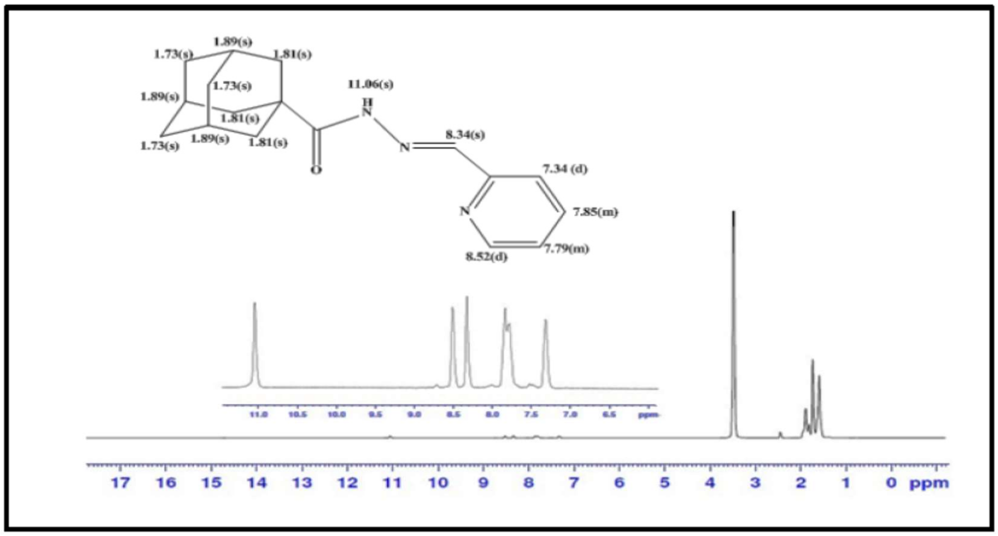

Figure S1. ^{1}H NMR spectrum of **APH**.

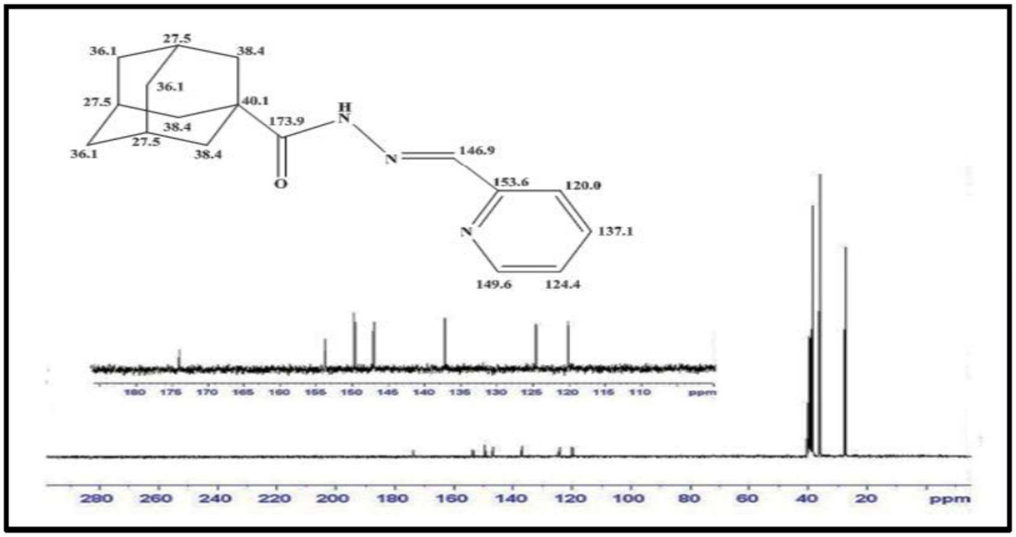

Figure S2. ^{13}C NMR spectrum of **APH**.

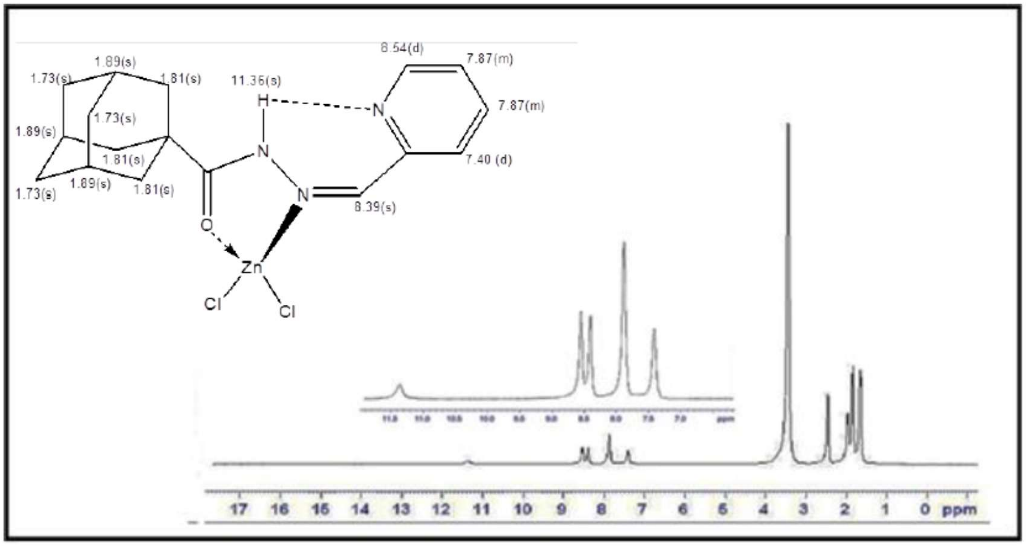

Figure S3. ^{1}H NMR spectrum of complex **4**.

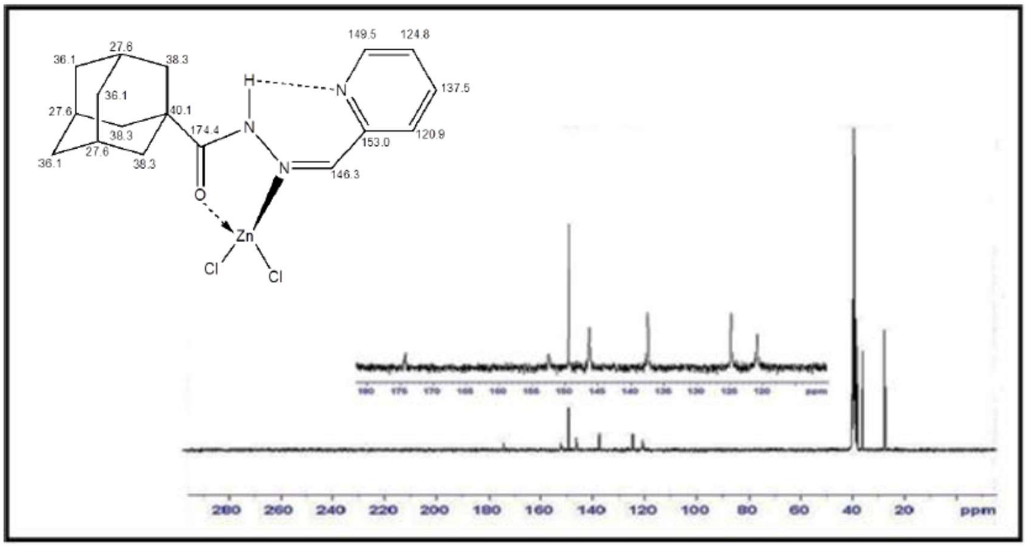

Figure S4. ^{13}C NMR spectrum of complex **4**.

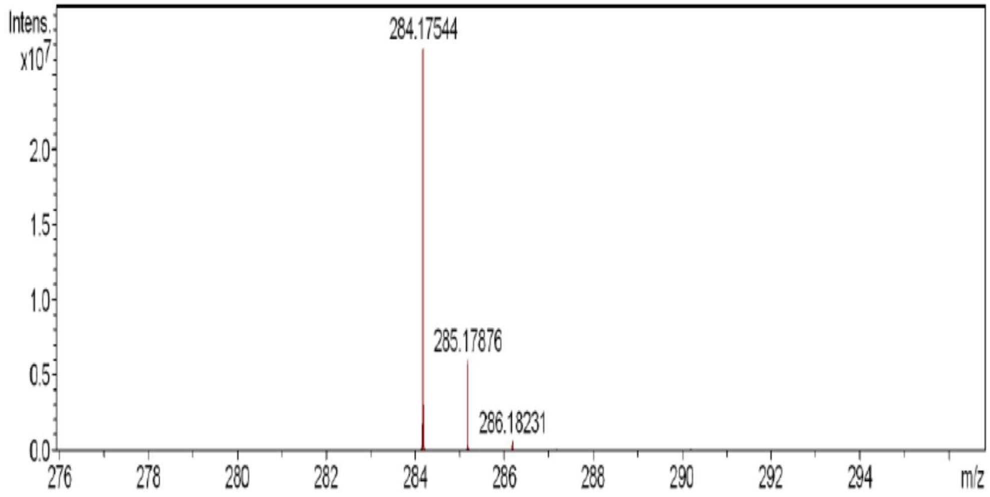

Figure S5. ESI-HRMS spectrum of **APH**.

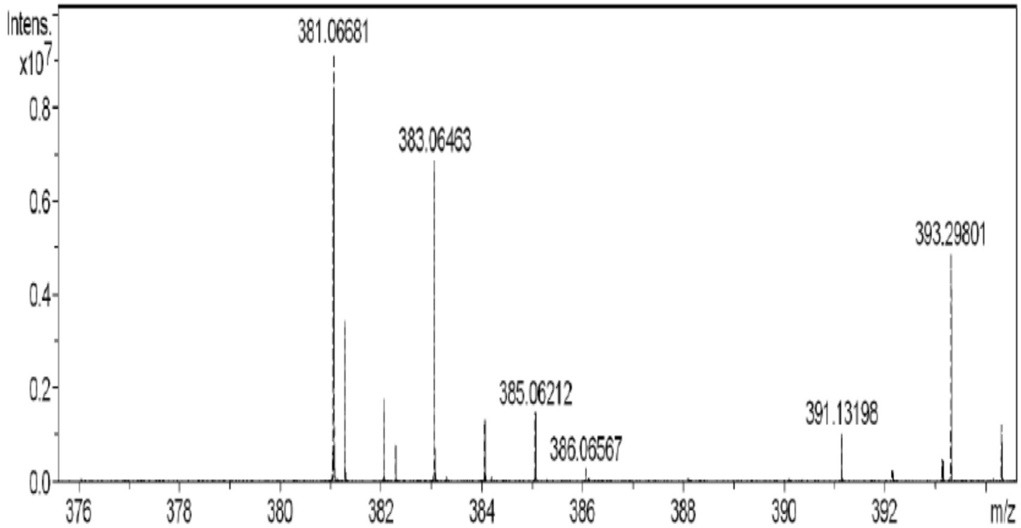

Figure S6. ESI-HRMS spectrum of complex **1**.

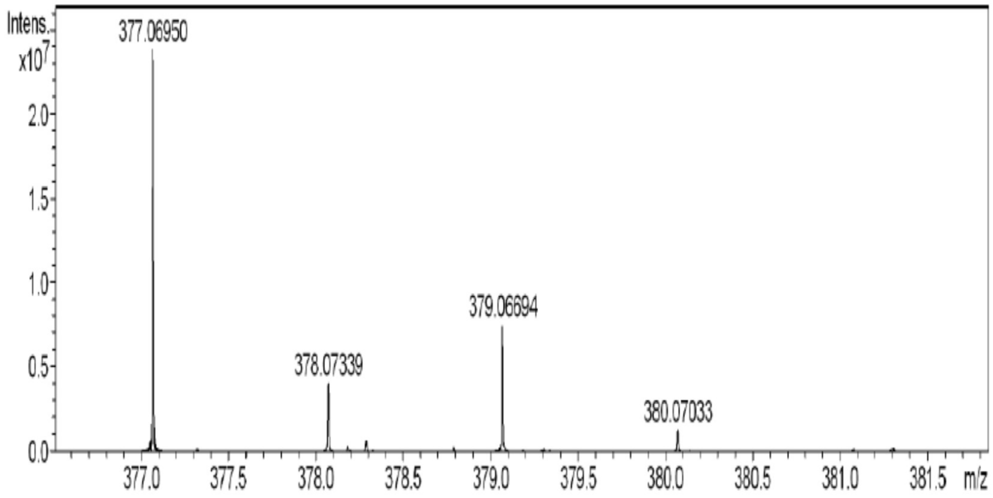

Figure S7. ESI-HRMS spectrum of complex **2**.

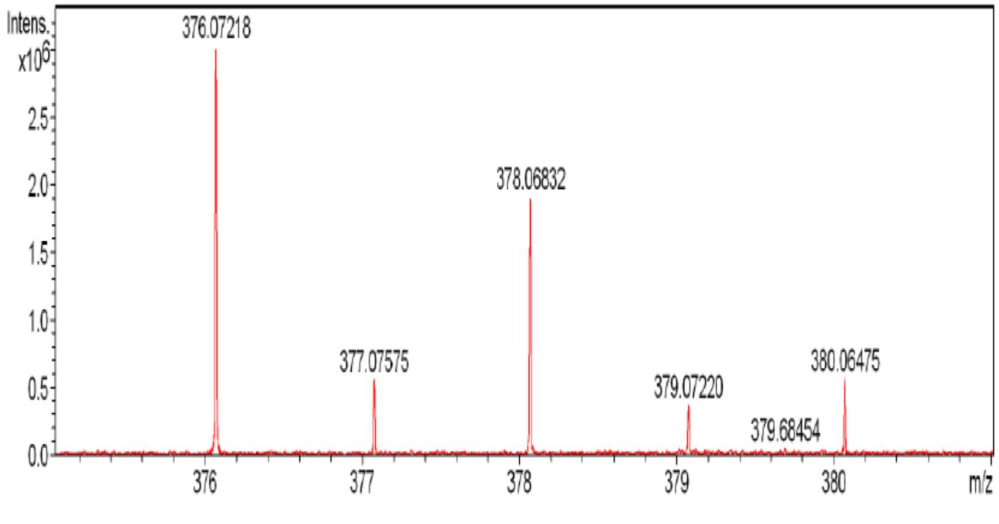

Figure S8. ESI-HRMS spectrum of complex **3**.

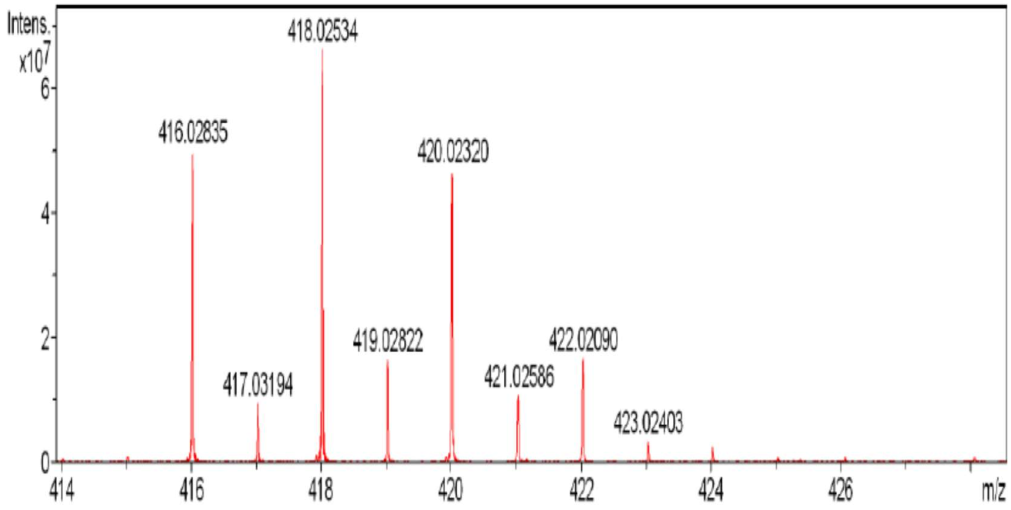

Figure S9. ESI-HRMS spectrum of complex **4**.

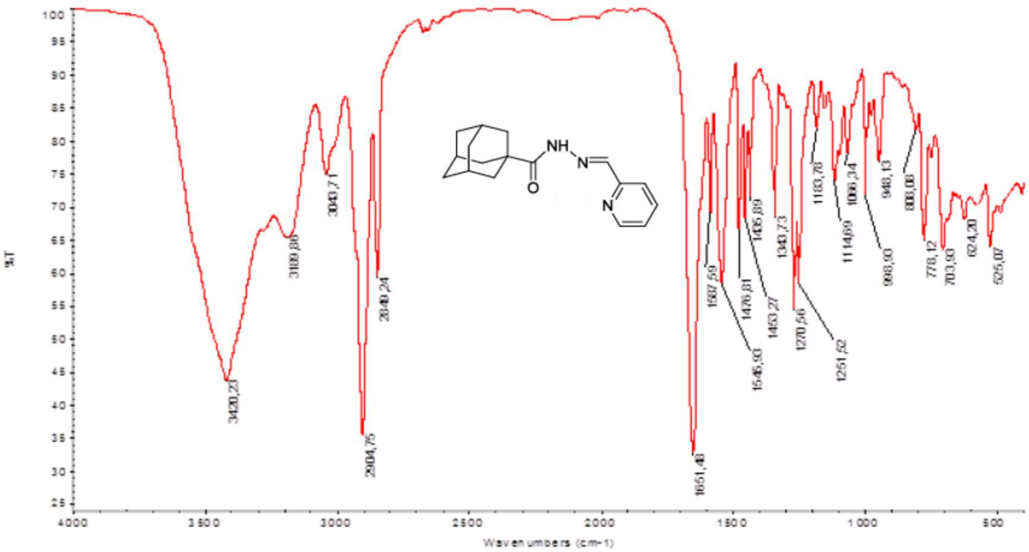

Figure S10. FT-IR spectrum of **APH**.

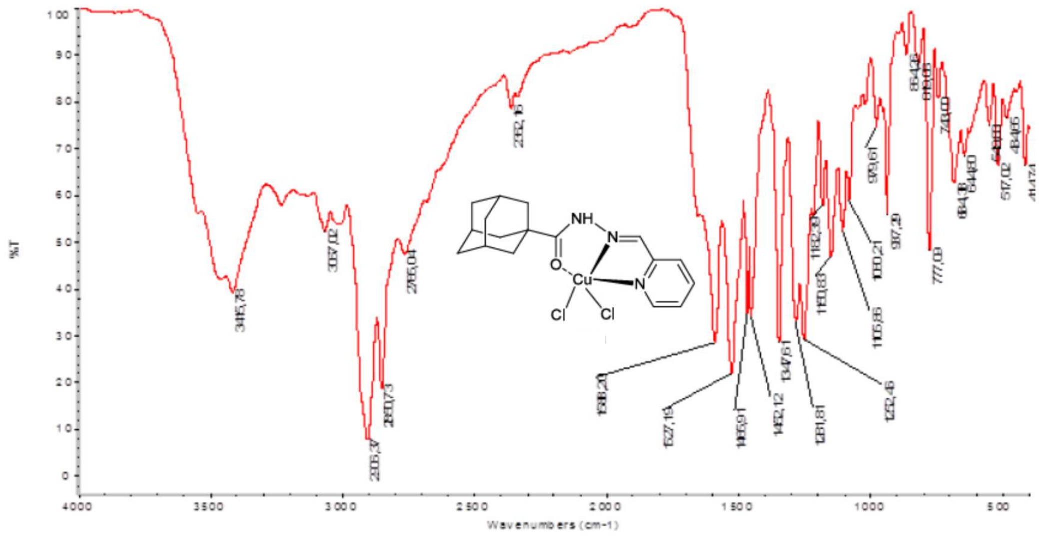

Figure S11. FT-IR spectrum of complex **1**.

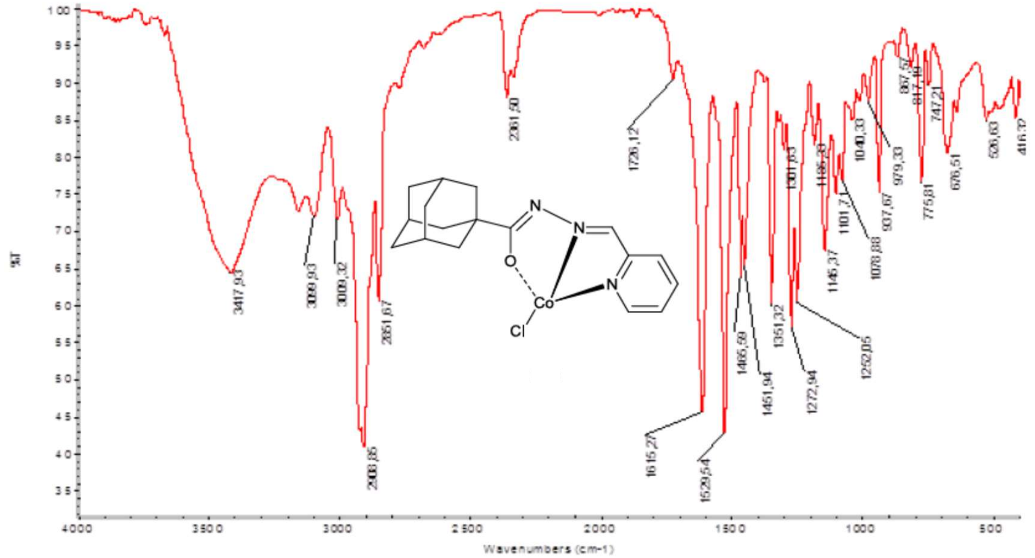

Figure S12. FT-IR spectrum of complex **2**.

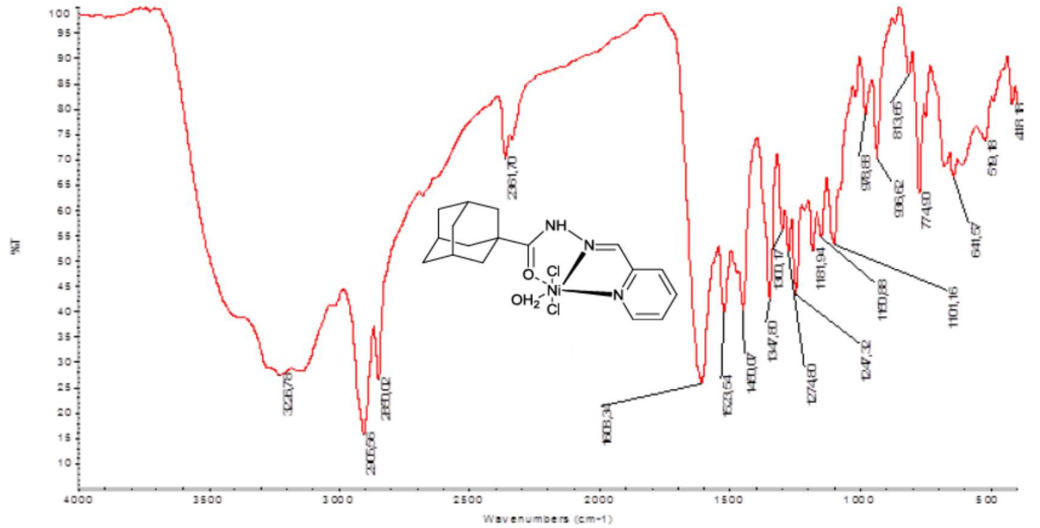

Figure S13. FT-IR spectrum of complex **3**.

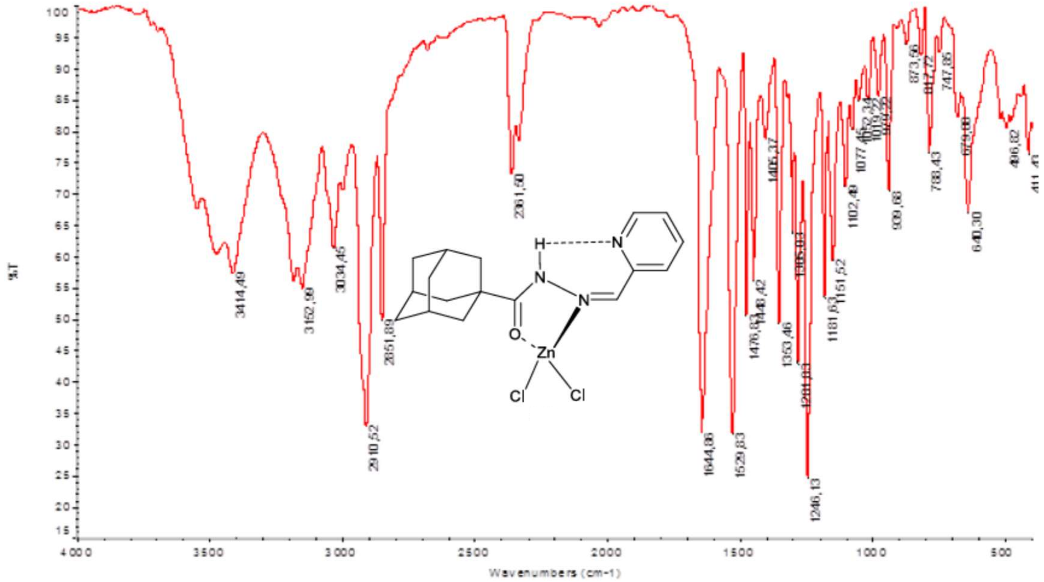

Figure S14. FT-IR spectrum of complex **4**.
